# Pre-surgical depression and anxiety and recovery following coronary artery bypass graft surgery

**DOI:** 10.1007/s10865-016-9775-1

**Published:** 2016-08-23

**Authors:** Lydia Poole, Amy Ronaldson, Tara Kidd, Elizabeth Leigh, Marjan Jahangiri, Andrew Steptoe

**Affiliations:** 1 Department of Epidemiology and Public Health, University College London, 1-19 Torrington Place, London, WC1E 6BT UK; 2 Department of Cardiac Surgery, St George’s Hospital, University of London, Blackshaw Road, London, SW17 0QT UK

**Keywords:** Depression, Anxiety, Coronary artery bypass graft surgery, Recovery, Major adverse cardiac events, Pain, Longitudinal study

## Abstract

We aimed to explore the combined contribution of pre-surgical depression and anxiety symptoms for recovery following coronary artery bypass graft (CABG) using data from 251 participants. Participants were assessed prior to surgery for depression and anxiety symptoms and followed up at 12 months to assess pain and physical symptoms, while hospital emergency admissions and death/major adverse cardiac events (MACE) were monitored on average 2.68 years after CABG. After controlling for covariates, baseline anxiety symptoms, but not depression, were associated with greater pain (β = 0.231, *p* = 0.014) and greater physical symptoms (β = 0.194, *p* = 0.034) 12 months after surgery. On the other hand, after controlling for covariates, baseline depression symptoms, but not anxiety, were associated with greater odds of having an emergency admission (OR 1.088, CI 1.010–1.171, *p* = 0.027) and greater hazard of death/MACE (HR 1.137, CI 1.042–1.240, *p* = 0.004). These findings point to different pathways linking mood symptoms with recovery after CABG surgery.

## Introduction

Co-morbid depression is commonly observed in patients suffering from coronary heart disease (Dickens 2015). Two landmark studies documented the impact of depression on survival following coronary artery bypass graft (CABG) surgery. Blumenthal and colleagues (Blumenthal et al. 2003) recruited 817 patients awaiting CABG and followed participants up for an average of 5.2 years. They found that patients with depression before CABG had increased risk of death from all causes compared with those without depression. Connerney and colleagues (Connerney et al. 2001) used standard diagnostic interviews in 309 patients in the days following CABG, assessing both current and history of depression. Patients were followed up at six and 12 months. They found that depression experienced after CABG was an independent risk factor for cardiac events after controlling for other risk factors including disease severity. A recent population study has also corroborated the effect of depression before CABG (Stenman et al. 2014) on mortality.

Pre- and post-operative depression symptoms have been shown to affect other indicators of recovery after CABG surgery. For example, Burg and colleagues (Burg et al. 2003) found that patients who were depressed prior to CABG had higher levels of medical complications during the six months following surgery, and were more likely to report poor quality of life and worse recovery. We recently reported that pre-operative depression symptoms were associated with longer post-operative hospital stays (Poole et al. 2014) following CABG surgery. Depressive symptoms following CABG have been associated with graft disease progression (Wellenius et al. 2008) and hospital readmissions (Tully et al. 2008). In addition, post-operative depression symptoms have been associated with poorer functional recovery such as shorter walking distances (Doering et al. 2005) and poorer physical recovery including more infections and impaired wound healing (Doering et al. 2005, 2008) in CABG patients.

A few studies have also implicated anxiety in predicting poorer prognosis in CABG patients. Tully and colleagues (Tully 2008) found that patients with higher levels of anxiety prior to CABG were at greater risk of mortality; these findings have been corroborated more recently (Tully et al. 2015). Another study has also confirmed the effect of anxiety following CABG on mortality (Rosenbloom et al. 2009), and Oxlad and colleagues showed anxiety in the immediate post-operative period was associated with greater hospital readmissions six months after CABG (Oxlad et al. 2006).

To date, only three studies have examined the combined effects of depression and anxiety symptoms in CABG patients to assess the relative importance of these mood disorders on recovery. However, there have been mixed findings. The first found pre-operative depression symptoms, but not anxiety symptoms, to predict poor health related quality of life in 193 participants followed up six months after surgery (Tully et al. 2009). The second included 158 CABG patients and found pre-operative generalised anxiety disorder, but not major depression, predicted major adverse cardiovascular and cerebrovascular events (Tully et al. 2015). The third found post-operative anxiety, but not depression symptoms, to predict major adverse events and mortality up to four years following surgery in a sample of 180 CABG patients (Székely et al. 2007). Therefore, it is not yet clear to what extent anxiety symptoms predict cardiac morbidity and mortality over and above depression symptoms or vice versa.

Since measures of mood are likely to be influenced by acute feelings of anticipation leading up to the surgical procedure, we chose to study the independent effects of depression and anxiety symptoms measured a month prior to surgery. In addition, in order to shed some light on the discrepant findings surrounding the role of depression and anxiety on later recovery, we assessed multiple recovery endpoints. Specifically, this study sought to explore the effects of pre-operative depression and anxiety symptoms on physical recovery following CABG to include sensory pain and physical symptom reporting, and emergency department admissions, and major adverse cardiac events (MACE) and/or death following CABG surgery.

## Method

### Participants

The study uses data collected in the Adjustment and Recovery after Cardiac Surgery (ARCS) Study (Poole et al. 2014a; Steptoe et al. 2015). Participants included in these analyses are the 251 CABG surgery patients (mean age: 67.91 ± 8.85 years, 13.1 % females) who provided complete data and who were recruited from a pre-surgery assessment clinic at St. George’s Hospital, London. The power analysis for this study was based on the paper by Connerney and colleagues (Connerney et al. 2001) and calculated using nQuery Advisor 4.0. Based on their findings an association of depression with clinical cardiac outcomes would be detected at 80 % power (two sided test, *p* < 0.05) with 64 patients with high and low depression scores. Using data from our pilot study we estimated a prevalence of 30 % of cases with high depression scores on the BDI prior to surgery, indicating a sample size of at least 213 would be needed to detect a significant effect on death/MACE. While ARCS was not specifically powered to assess anxiety, using data from Szekely et al. (Székely et al. 2007) an association of anxiety with clinical cardiac outcomes would be detected at 80 % power (two sided test, *p* < 0.05) with 56 patients with high and low anxiety scores. Using data from our pilot study we estimated a prevalence of 25 % of cases with high anxiety scores on the HADS prior to surgery, indicating a sample size of at least 224 would be needed to detect a significant effect on death/MACE. Since our analyses were powered to investigate death/MACE, our analyses using the alternative outcomes are exploratory in nature.

Compared to the participants who were included in these analyses, the included participants were more likely to be male (x^2^ = 6.867, *p* = 0.008) and have one or more comorbidities (x^2^ = 46.055, *p* < 0.001), but otherwise did not differ on any other clinical or demographic variable. The baseline assessment took place on average 30 days before patients’ surgery and included measures of depression and anxiety symptoms and demographic measures completed by postal questionnaire. The follow-up assessment of self-reported outcomes, i.e. pain and symptom reporting, took place on average 397 days after CABG also by postal questionnaire. Clinical outcomes, i.e. death/MACE and emergency department admissions were collected on average 2.68 years (range: 1.84–3.44 years) after CABG. Inclusion criteria permitted only patients who were undergoing elective CABG surgery or CABG plus valve replacement to participate. CABG surgery included both on-pump and off-pump surgical procedures. In addition, participants had to be able to complete the questionnaires in English, and be 18 years or older. All procedures were carried out with the written consent of the participants. Ethical approval was obtained from the South West London research ethics committee.

### Measures

#### Predictor variables: depression and anxiety

The Beck Depression Inventory (BDI) (Beck et al. 1988) was used to measure depression symptoms at baseline. It is a 21-item questionnaire which asks the respondent to reflect on how they have been feeling over the past two weeks. Ratings were summed, with higher scores indicating greater emotional disturbance, with a range of 0–63 (Cronbach’s α = 0.85). For illustration purposes, we used a standard cut-off of </≥10 to indicate no depression and mild to severe depression respectively.

The Hospital Anxiety and Depression Scale (HADS) is a self-report measure of anxiety and depression for use in outpatient clinical settings (Zigmond and Snaith 1983). Only the 7-item anxiety scale was administered at baseline, capturing the extent to which each symptom has been experienced over the past two weeks. Items were summed to generate an overall score, with higher scores indicating greater anxiety (Cronbach’s α = 0.88).

#### Outcome variables: pain, physical symptoms, emergency department admissions, death/MACE

The McGill Pain Questionnaire-Short Form (MPQ-SF) (Melzack 1987) was developed as a brief version of the standard MPQ and is suitable for use in post-surgical patients. This measure was administered 12 months post-CABG. The sensory pain score was used here and reflects the sum of the intensity values for the sensory descriptor words (throbbing, aching, stabbing etc.). Higher scores indicate greater pain (sensory pain Cronbach’s α = 0.91).

The Coronary Revascularisation Outcomes Questionnaire (CROQ) (Schroter and Lamping 2004) was designed to assess quality of life and health outcomes following cardiac surgery. This study used the adverse effects subscale from the version adapted for CABG surgery and was administered 12 months after surgery. Patients were asked to rate the extent to which they had experienced a series of physical symptoms related to their surgery such as bruising, numbness and tingling and swelling, using a 5-point Likert scale ranging from 0 (Not at all) to 4 (A lot); higher scores indicate greater negative symptoms (Cronbach’s α = 0.81).

Post-operative MACE included admissions for myocardial infarction, unstable angina, stroke, and/or heart failure. Occurrence of MACE or death were combined to create a binary variable. Emergency department admissions were also categorised into no admissions or ≥1 admission. Mortality, MACE and emergency department admission data were gathered by reviewing in-hospital electronic and paper patient records.

#### Covariates: clinical and sociodemographic measures

Cardiovascular history, clinical factors during admission and management were also obtained from clinical notes. Clinical risk was assessed using the European System for Cardiac Operative Risk Evaluation (EuroSCORE) (Roques et al. 2003). EuroSCORE is a composite measure of procedural mortality risk based on 17 factors comprising patient-related factors (e.g. age, sex), cardiac-related factors (e.g. unstable angina, recent MI) and surgery-related factors (e.g. surgery on thoracic aorta). Items were scored in accordance with the ‘logistic EuroSCORE’ method to generate a percentage mortality risk estimate; further details of the scoring method can be found on the EuroSCORE website (www.euroscore.org/logisticEuroSCORE.htm) Goldstone (n.d.). In addition, the number of grafts a participant received and whether they underwent cardiopulmonary bypass (yes/no) were also recorded. History of diabetes was also taken from medical notes.

Participants were asked about any longstanding illnesses prior to surgery; responses were counted to compute a chronic illness burden variable to capture the number of illnesses a participant had in addition to their coronary artery disease. Participants also self-reported use of medications to include the use of antidepressants and anxiolytics. Smoking was measured as a binary variable at baseline (current smoker/non-smoker). Body mass index (BMI) was assessed at the pre-operative clinic appointment and calculated using the standard formula (kg/m^2^).

### Statistical analysis

Missing data (n = 75) on the self-reported outcomes meant that some analyses were performed on a reduced sample size. Of all participants, sensory pain and CROQ scores were available for 176 participants. Descriptive statistics describe the 251 participants who provided full baseline and clinical outcome data. Associations between variables were assessed using Pearson’s correlations for continuous data and independent t-tests or Chi-square tests for categorical variables. The predictor variables, depression and anxiety symptoms, were entered simultaneously into models since the assumption of no mulitcollinearity was not violated. Similar findings were found entering depression and anxiety symptoms separately into models. To test the association between baseline depression and anxiety symptoms on sensory pain and physical symptom reporting (CROQ), we used multiple linear regression analyses. We controlled for several potential confounders of the associations, namely BMI, smoking status, diabetes, chronic illness burden, use of cardiopulmonary bypass, EuroSCORE, and use of antidepressants and anxiolytics. Age and sex are included in EuroSCORE so were not entered separately to avoid double adjustment. The association between depression and anxiety symptoms and emergency department admissions were modelled using logistic regressions. Results are presented as adjusted odds ratios (OR) with 95 % confidence intervals (CI). The association between depression and anxiety symptoms and death/MACE were modelled using Cox proportional hazards regressions. The assumption of proportional hazards was upheld in all the models. Results are presented as adjusted hazard ratios (HR) with 95 % CI. Due to the small event rate in the emergency department admission and death/MACE analyses, we only controlled for EuroSCORE and chronic illness burden in these analyses to avoid over-fitting the model. We illustrated the significant associations by comparing the risk of death/MACE in patients with low/high depression (BDI </≥10), after adjustment for covariates. Secondary analyses were performed for all models in which the mean-centred depression-anxiety symptom interaction term was included as a covariate. No significant interactions were found so the results are not reported here. All analyses were conducted using SPSS version 21. Two-tailed tests were used throughout and the significance level was set at *p* < 0.05, though exact significance levels are reported.

## Results

Descriptive characteristics of the sample are displayed in Table 1. The majority of participants were male (87.0 %), overweight (BMI >25 = 82.5 %) and of White ethnic origin (88.0 %). Predicted mortality risk according to EuroSCORE ranged from 1.5 % to 22.0 %. Comorbidities were common, particularly diabetes which was present in a quarter of participants and hypertension (79.3 %). The majority of participants underwent on-pump surgery in isolation. Depression and anxiety symptoms were prevalent at baseline. Over a third (35.9 %) of participants reported moderate to severe depression symptoms (BDI scores ≥10) and just under a third of participants (31.9 %) reported anxiety symptoms within the mild to severe range (HADS score ≥8). Depression and anxiety symptoms were significantly correlated (*r* = 0.641, *p* < 0.001). Twelve-months following CABG surgery approximately half (48.9 %) of participants were still experiencing some pain, and 90.3 % had physical symptoms relating to their surgery. Sensory pain and physical symptom reporting were positively correlated (*r* = 0.690*, p* < 0.001). With regards to the clinical outcomes there were 19 death/MACE events of which 9 participants died, 8 participants experienced a MACE, and a further 2 participants experienced both MACE and death.

**Table 1:** Demographic, clinical and mood characteristics of the sample (N = 251)

Characteristic	Mean ± SD or N (%)
*Demographics*	
Age (years)	67.91 ± 8.85
Female	33 (13.1)
BMI (kg/m^2^)	28.84 ± 4.33
Married/cohabiting	196 (75.7)
Ethnicity–white British/other white	221 (88.0)
Smoker	20 (8.0)
*Co*-*morbidities*	
Chronic illness burden	0.47 ± 0.65
Diabetes	63 (25.0)
Hypertension	199 (79.3)
Pulmonary disease	18 (7.2)
Neurological disorder	19 (8.0)
Extracardiac arteriopathy	22 (8.8)
*Clinical factors*	
Logistic EuroSCORE (%)	4.52 ± 3.24
MI <30 days prior to CABG	1 (<0.0)
CABG in isolation	191 (76.1)
Number of grafts	2.96 ± 1.12
On-pump	200 (79.7)
Sensory pain (MPQ-SF) 12-months post-CABG	1.75 ± 3.74*
Physical symptoms (CROQ) 12-months post-CABG	5.68 ± 5.50*
Emergency department admissions ≥1	23 (9.2)
Death/MACE ≥1	19 (7.6)
*Mood factors*	
Baseline BDI score	8.62 ± 6.57
Baseline HADS-anxiety score	5.96 ± 4.30
Antidepressant use	14 (5.6)
Anxiolytic use	2 (0.8)

* N = 176

### Baseline depression and anxiety symptoms predicting pain and physical symptoms 12 months after CABG

Table 2 displays the regression model examining the prospective association between pre-operative depression and anxiety symptoms and sensory pain measured 12 months after CABG surgery. The results show that greater anxiety symptoms were associated with greater post-operative pain (β = 0.215, *p* = 0.023), after controlling for covariates. Baseline depression was not a significant predictor in this model. The model accounted for 8.5 % of variance in sensory pain.

**Table 2:** Multiple regression showing baseline anxiety predicting sensory pain (N = 176)

Model	*B*	*SE*	*β*	95 % CI	*p*
Baseline BDI score	0.065	0.059	0.105	−0.052–0.183	0.272
Baseline HADS-anxiety score	0.192	0.083	0.215	0.027–0.356	0.023
BMI	−0.051	0.073	−0.054	−0.194–0.093	0.487
Smoker	−1.207	1.161	−0.082	−3.499–1.085	0.300
Diabetes	0.582	0.956	0.064	−1.306–2.469	0.544
Chronic illness burden	0.902	0.617	0.154	−0.315–2.119	0.145
Cardiopulmonary bypass	−0.598	0.691	−0.064	−1.963–0.766	0.388
EuroSCORE	−0.074	0.094	−0.061	−0.259–0.111	0.431
Baseline anxiolytic use	−0.570	2.681	−0.016	−5.863–4.723	0.832
Baseline antidepressant use	2.121	1.320	0.125	−0.486–4.727	0.110

Table 3 displays the regression model examining the prospective association between pre-operative depression and anxiety symptoms and physical symptoms measured 12 months after CABG surgery. The results show that greater anxiety symptoms were associated with greater physical symptoms (β = 0.191, *p* = 0.035), after controlling for covariates. Baseline depression was not a significant predictor in this model. The model accounted for 15.1 % of variance in physical symptoms.

**Table 3:** Multiple regression showing baseline anxiety predicting physical symptoms (N = 176)

Model	*B*	*SE*	*β*	95 % CI	*p*
Baseline BDI score	0.149	0.083	0.164	−0.015–0.313	0.075
Baseline HADS-anxiety score	0.247	0.117	0.191	0.017–0.478	0.035
BMI	0.134	0.102	0.098	−0.066–0.335	0.188
Smoker	−0.939	1.625	−0.044	−4.148–2.269	0.564
Diabetes	−0.454	1.338	−0.034	−3.097–2.188	0.735
Chronic illness burden	2.069	0.863	0.243	0.365–3.773	0.018
Cardiopulmonary bypass	0.410	0.967	0.030	−1.500–2.319	0.673
EuroSCORE	0.103	0.131	0.058	−0.156–0.363	0.432
Baseline anxiolytic use	−8.039	3.753	−0.157	−15.448–0.629	0.034
Baseline antidepressant use	4.554	1.848	0.185	0.905–8.203	0.015

### Baseline depression and anxiety symptoms predicting clinical outcomes after CABG

Table 4 displays models showing the relationship between baseline depression and anxiety measures and post-operative emergency department admissions. The results show that after controlling for chronic illness burden and EuroSCORE, a 1-point increment on the BDI was associated with an 8.8 % increase in the risk of having an emergency department admission (OR 1.088, CI 1.010-1.171, *p* = 0.027). Anxiety symptoms were not significantly associated with increased risk of emergency department admissions.

**Table 4:** Logistic regression showing baseline depression predicting emergency department admissions (N = 251)

Model	*OR*	95 % CI	*p*
Baseline BDI score	1.088	1.088–1.171	0.027
Baseline HADS-anxiety score	1.003	1.003–1.171	0.966
Chronic illness burden	1.193	1.193–2.309	0.601
EuroSCORE	1.087	1.087–1.221	0.158

**Fig. 1:**
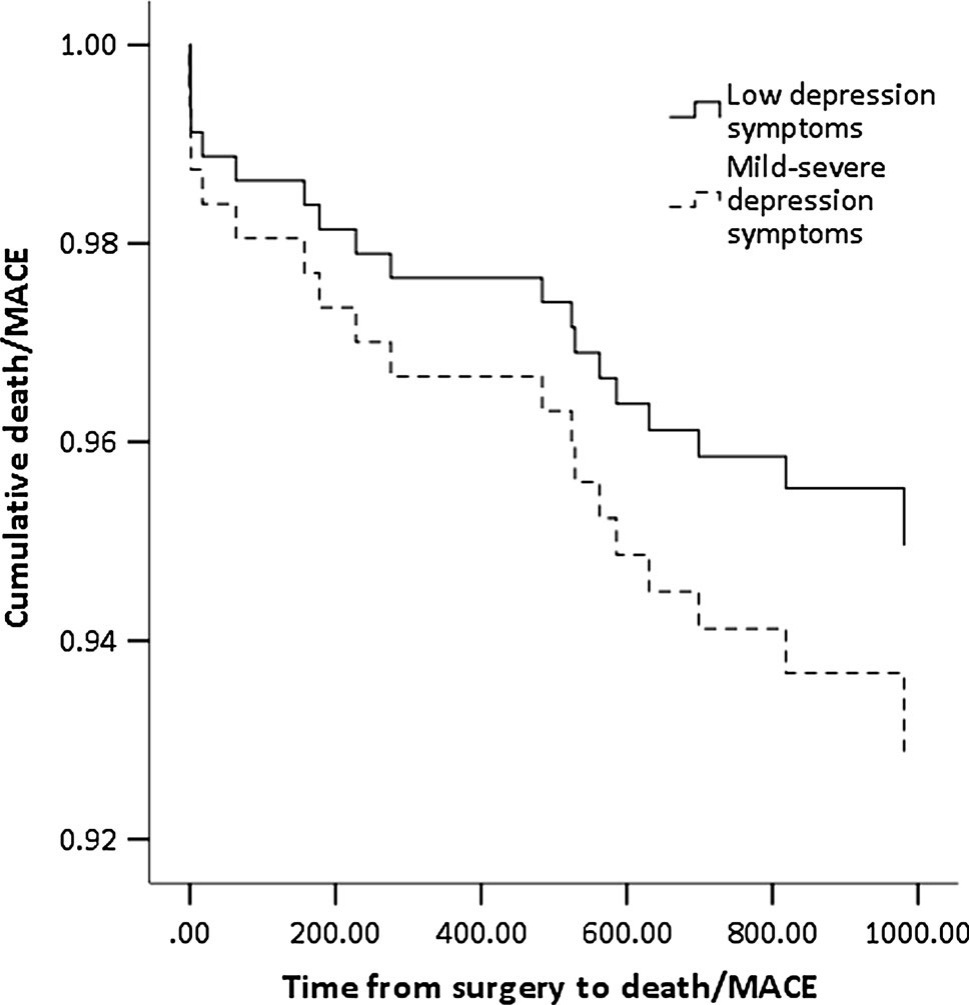
Death/MACE by depression severity status on the BDI; fully adjusted model (N = 251)

Table 5 shows the results from the Cox regression model for depression and anxiety symptoms predicting death/MACE following CABG. Results show that a 1-point increment on the BDI was associated with a 13.7 % increased hazard of death/MACE after controlling for covariates (HR 1.137, CI 1.042–1.240, *p* = 0.004). Anxiety symptoms were not significant predictors in this model. This association is illustrated in Fig. 1, showing the risk of death/MACE in patients with low/high depression (BDI </≥10), after adjustment for covariates.

**Table 5:** Cox regression showing baseline depression predicting death/MACE (N = 251)

Model	*HR*	95 % CI	*p*
Baseline BDI score	1.137	1.042–1.240	0.004
Baseline HADS-anxiety score	0.871	0.748–1.013	0.073
Chronic illness burden	1.609	0.859–3.016	0.138
EuroSCORE	1.231	1.113–1.361	<0.001

## Discussion

This study sought to examine the effects of depression and anxiety on surgical recovery in a sample of patients undergoing first-time, elective CABG. Depression and anxiety were entered simultaneously into models in order to ascertain the effect of depression over and above anxiety (and vice versa) on predicting recovery. We showed that pre-operative anxiety predicted the self-reported outcomes, sensory pain and physical symptoms 12 months after surgery, while pre-operative depression predicted the objective outcomes, emergency department admission and death/MACE in the years after surgery.

Our findings can be interpreted in light of previous research in this field. Multiple studies have shown depression prior to CABG to be a significant predictor of morbidity and mortality in the months and years following surgery (Blumenthal et al. 2003; Burg et al. 2003; Burg et al. 2003), and similar effects have also been observed for anxiety (Tully et al. 2008; Tully et al. 2015). Interestingly in our study we found that anxiety symptoms predicted the self-reported measures whereas depression symptoms predicted the objective measures.

We surmise that in our study greater anxiety symptoms increased the likelihood of patients being hypervigilant to somatic symptoms long after the acute phase of recovery had passed. Previous work has supported the association between anxiety and over-reporting of somatic symptoms (Hoehn-Saric et al. 2004). Indeed, health anxiety has a lifetime prevalence of approximately 5.7 % (Sunderland et al. 2013) and is a well-established contributing factor to somatosensory amplification (Barsky and Wyshak 1990). Health anxiety has been found to detrimentally affect wellbeing, quality of life and use of healthcare services (Asmundson et al. 2010; Barsky et al. 2001), but is responsive to cognitive behaviour therapy (Tyrer et al. 2014). A meta-analysis investigating the association of depression and anxiety with medical symptom reporting suggests that both depression and anxiety were at least as strongly associated with somatic symptoms as objective physiological measures of illness (Katon et al. 2007). In addition, Katon proposes that depression and anxiety prevent habituation to the symptoms of physical illnesses, such as pain (Katon 2003). In our study, we did not support the role of depression symptoms on symptom burden, perhaps because unlike the studies cited above, we considered depression and anxiety simultaneously in models.

In our study we also found individuals with greater depression symptoms were more likely to experience a verifiable clinical episode. These results suggest a different mechanism was in operation in those with depression symptoms compared to those with anxiety symptoms. Multiple mechanisms have been proposed linking depression to adverse events in cardiac patients including biological, social and behavioural factors. We have previously shown that heightened C-reactive protein levels in response to CABG surgery mediated the association between depression and longer hospital stays (Poole et al. 2014b). In addition we have also shown that greater interferon-gamma responses to surgery were associated with increased risk of depression 12 months after CABG surgery (Steptoe et al. 2015). Therefore, inflammation presents one possible pathway linking depression and greater adverse events in our sample. We have also shown that social factors such as low socioeconomic status (Poole et al. 2014) and behavioural factors such as sleep (Poole et al. 2014a) are also important for outcomes in CABG patients. Medication non-adherence could also be another explanatory factor, since depressed patients have been shown to be less adherent to cardiac medications than their non-depressed counterparts (Rieckmann et al. 2006, 2011). It is possible that depression and anxiety symptoms differentially affected recovery in the ARCS participants by operating along different mechanistic pathways. More work would be needed to tease out the exact causes of these effects using a larger sample of participants.

We considered depression and anxiety simultaneously in models, which we believe a more ecologically valid approach. Depression and anxiety often present as co-morbid conditions, which may partly be explained by the common symptom criteria for both disorders. Theories have been proposed in which anxiety and depressive disorders should be viewed collectively as part of a higher-order diagnosis of negative affectivity, characterised by heightened levels of distress, negative emotionality and neuroticism (Andrews et al. 2009; Brown and Barlow 2009; Prenoveau et al. 2010; Watson 2009). However, a recent paper by Tully found that a clinical diagnosis of generalised anxiety disorder was the best predictor of MACE after CABG surgery than other classifications of mood disorders and negative affect symptom clusters (Tully et al. 2015). In a sample of patients with stable coronary artery disease, Frasure-Smith and Lespérance (2008) reported that patients with comorbid elevated anxiety and depression symptoms were not at elevated risk of MACE, compared to those with one condition alone. In contrast, we found depression symptoms, but not anxiety symptoms to predict death/MACE after CABG surgery. The discrepancy between ours and Tully’s findings could partly be explained by the difference in the sample. We recruited first-time, elective CABG patients to the ARCS study whereas Tully and colleagues also included emergency patients. This difference in demographic is reflected in the number of previous myocardial infarctions experienced by participants. Only one of our participants experienced a myocardial infarction in the month preceding surgery compared with just over one-third of participants in the Tully study. Therefore there are likely differences between our studies regarding the extent to which anxiety brought on by this major adverse cardiac event was captured.

Our findings are thought to be clinically relevant since intervention studies could help ameliorate negative mood symptoms in CABG patients. Intervention studies designed to improve emotional wellbeing have generally focussed on post-CABG depression, and have shown mixed results. Positive results were found by Freedland and colleagues (Freedland et al. 2009) who conducted a 12 week randomised controlled trial to assess the efficacy of two different types of non-pharmacological treatment for depression symptoms after CABG, in comparison to usual care controls. Results showed significantly higher rates of remission in depressed patients who received cognitive behavioural therapy or supportive stress-management compared to usual care controls, at three- and nine-month follow-up. The Bypassing the Blues randomised controlled trial, investigated the impact of a telephone delivered collaborative care intervention in post-CABG depressed patients, compared to usual care depressed controls and non-depressed controls. The intervention group received a tailored nurse-led telephone delivered programme for up to eight months following CABG. Results showed significant improvements in health-related quality of life and mood in the intervention group compared to usual care controls, however the effect was stronger for men than women (Rollman et al. 2009; Rollman and Herbeck Belnap 2011). Despite these studies demonstrating a positive impact on emotional wellbeing, three other randomised controlled trials have been less conclusive (Furze et al. 2009; Lie et al. 2007; Sebregts et al. 2005). As yet no trials have shown treatment of depression translates to improved clinical outcomes; however, positive effects were reported in the SUPRIM study (Gulliksson et al. 2011) of stress management and these findings could potentially inform future depression trials. In addition, more work is needed to assess the benefits of treating depression and anxiety prior to surgery.

There are several strengths and weakness to our study. In terms of strengths, the longitudinal design of the ARCS study allows for the temporal relationship between depression, anxiety and surgical recovery to be analysed. Moreover, our collection of both self-reported and clinical endpoints has allowed us to assess several indicators of recovery. Baseline assessments of mood took place a month prior to surgery, minimising the effects of acute distress in the anticipation of major surgery. In addition, the ARCS study examined patients undergoing CABG at a single hospital and therefore removes the influence of inter-hospital variation in patient care. There are also some weakness that must be borne in mind. Firstly, we have relied on questionnaire measures of mood, which restricts us from generalising our results to clinically depressed and anxious samples. The validity of the HADS has recently been questioned, with concerns regarding its latent structure (Cosco et al. 2012; Coyne and van Sonderen 2012). However, others have found in favour of its suitability, and it has just been listed as among the acceptable measures for screening for depression in adults described by the US Preventive Services Task Force (Siu et al. 2016). The HADS-anxiety score was used as a continuous measure in our models to sidestep the issue of caseness. Secondly, we only had a small number of death/MACE clinical events in our study so were unable to control for a wide range of clinical and demographic confounders in the analyses. The findings we have reported may have clinical implications regarding the screening and management of depression and anxiety in a CABG population.

In conclusion, we found that greater pre-CABG anxiety symptoms were associated with poorer self-reported outcomes 12 months after surgery, including greater pain and physical symptoms. On the other hand, greater pre-CABG depression symptoms were associated with the objective clinical outcomes: death/MACE and emergency department admissions in the years following surgery. Further work is needed to delineate the pathways of these effects and the most appropriate treatment strategies.

## Abbreviations

ARCS study: Adjustment and recovery after cardiac surgery study
BDI: Beck depression inventory
BMI: Body mass index
CABG: Coronary artery bypass graft
CI: Confidence intervals
EuroSCORE: European system for cardiac operative risk evaluation
HADS: Hospital anxiety and depression scale
HR: Hazard ratio
OR: Odds ratio
